# In vitro and in vivo characterization of anti-malarial acylphenoxazine derivatives prepared from basic blue 3

**DOI:** 10.1186/s12936-019-2873-0

**Published:** 2019-07-15

**Authors:** Takahiro Tougan, Kazunori Takahashi, Mayumi Ikegami-Kawai, Masako Horiuchi, Shiho Mori, Maiko Hosoi, Toshihiro Horii, Masataka Ihara, Masayoshi Tsubuki

**Affiliations:** 1 Department of Molecular Protozoology, Research Institute for Microbial Diseases, Osaka University, 3-1 Yamadaoka, Suita, Osaka 565-0871 Japan; 2 Institute of Medicinal Chemistry, Hoshi University, 2-4-41 Ebara, Shinagawa, Tokyo 142-8501 Japan; 3 Faculty of Pharmaceutical Sciences, Hoshi University, 2-4-41 Ebara, Shinagawa, Tokyo 142-8501 Japan

**Keywords:** Basic blue 3, ITT derivatives, Anti-malarial activity, Cytotoxicity, π-Delocalized lipophilic cations, DLC hypothesis, Mitochondria, Malaria, *Plasmodium*, An automated haematology analyzer (XN-30)

## Abstract

**Background:**

Basic blue 3 is a promising anti-malarial lead compound based on the π-delocalized lipophilic cation hypothesis. Its derivatives with nitrogen atoms bonded to carbon atoms at the 3- and 7-positions on the phenoxazine ring were previously shown to exert potent antiprotozoal activity against *Plasmodium falciparum*, *Trypanosoma cruzi*, *Trypanosoma brucei rhodesiense*, and *Leishmania donovani* parasites in vitro. However, compounds with nitrogen modification at the 10-position on the phenoxazine ring were not evaluated.

**Methods:**

Six acylphenoxazine derivatives (ITT-001 to 006) with nitrogen modification at the 10-position on the phenoxazine ring, which were synthesized from basic blue 3, were characterized and evaluated for anti-malarial activity in vitro with an automated haematology analyzer (XN-30) and light microscopy. Intensity of self-fluorescence was measured using a fluorometer. Localization of basic blue 3 was observed by fluorescence microscopy. Cytotoxicity was evaluated using human cell lines, HEK293T and HepG2 cells. Finally, anti-malarial activity was evaluated in a rodent malaria model.

**Results:**

All the six derivatives showed anti-malarial efficacy even against chloroquine-, pyrimethamine-, and artemisinin-resistant field isolates similar to the sensitive strains and isolates in vitro. The efficacy of basic blue 3 was the strongest, followed by that of ITT-001 to 004 and 006, while that of ITT-005 was the weakest. Basic blue 3 showed strong self-fluorescence, whereas ITT derivatives had five- to tenfold lower intensity than that of basic blue 3, which was shown by fluorescence microscopy to be selectively accumulated in the plasmodial cytoplasm. In contrast, ITT-003, 004, and 006 exhibited the lowest cytotoxicity in HEK293T and HepG2 cells in vitro and the highest selectivity between anti-malarial activity and cytotoxicity. The in vivo anti-malarial assay indicated that oral administration of ITT-004 was the most effective against the rodent malaria parasite, *Plasmodium berghei* NK65 strain.

**Conclusions:**

The six ITT derivatives were effective against chloroquine- and pyrimethamine-resistant strains and artemisinin-resistant field isolates as well as the sensitive ones. Among them, ITT-004, which had high anti-malarial activity and low cytotoxicity in vitro and in vivo, is a promising anti-malarial lead compound.

**Electronic supplementary material:**

The online version of this article (10.1186/s12936-019-2873-0) contains supplementary material, which is available to authorized users.

## Background

Emergence of anti-malarial drug-resistant *Plasmodium falciparum* parasite is an obstacle in the fight against malaria [1]. In 2006, the World Health Organization (WHO) recommended artemisinin (ART)-based combination therapy (ACT) as the first-line treatment for uncomplicated *P. falciparum* [2]. Unfortunately, ART-resistant parasites have emerged in Southeast Asia (SEA) and have subsequently spread across several neighboring countries to the Greater Mekong Subregion of SEA in recent years [3–9]. Moreover, the emergence of ART-resistant indigenous isolates in Africa was reported in 2017, which would be a concern for future treatment, since suitable replacement drugs are limited [10]. Therefore, anti-malarial drugs with efficacy against ART-resistant parasites are required.

Previous studies reported novel antiprotozoal compounds based on the π-delocalized lipophilic cations (DLC) hypothesis [11–17], in which hydrophobic cations containing delocalized π-electrons accumulate in the parasite mitochondria and inhibit metabolic activity [18]. Among them, phenoxazinium salt (basic blue 3 in this study) displayed strong antiprotozoal activity with high selectivity [17]. However, for their synthesis, the reactions of *m*-aminophenols and *p*-nitrosoanilines in perchloric acid produces very poor yields (2–40%) [17]. Moreover, these compounds are unsuitable for clinical use because they are typically purified using zinc chloride [19]. Therefore, a new method was developed to obtain these compounds with high purity via zinc chloride-free chromatography followed by crystallization [11]. In addition, some of their derivatives in which nitrogen atoms are bonded to carbon atoms at the 3- and 7-positions on the phenoxazine ring were modified and showed potent antiprotozoal activity against *P. falciparum*, *Trypanosoma cruzi*, *Trypanosoma brucei rhodesiense*, and *Leishmania donovani* parasites in vitro [20]. Furthermore, benzo[*a*]phenoxazine (SSJ-183) was developed and its high anti-malarial efficacy was demonstrated in vitro and in vivo [12, 21]. Moreover, it was discovered that the derivatives have a potential application as reversible near-infrared pH sensors [22].

The automated haematology analyzer (XN-30) (Sysmex, Kobe, Japan) detects parasite-infected red blood cells (iRBCs) in approximately 1 min by applying a technique similar to that used in flow cytometry and measures 10 haematological parameters, including the total RBC counts, using a sheath flow direct count. Furthermore, the XN-30 analyzer has been equipped with an algorithm for in vitro cultured parasites to accurately calculate parasitaemia as well as differentiate and quantify the developmental stages of parasites [23]. From these analyses, the XN-30 analyzer objectively evaluates and characterizes the efficacy of anti-malarial compounds by calculating the half-maximal (50%) inhibitory concentration (IC_50_) and classifying anti-malarial efficacy into four groups [24]. This technology was used to better characterize the outcomes in this study.

In the current study, six acylphenoxazine derivatives (termed ITT derivatives; ITT-001 to 006) were synthesized. The derivatives were characterized and evaluated for anti-malarial activity in vitro and in vivo. The analyses suggested that ITT-004 had the highest therapeutic potential against malaria.

## Methods

### Synthesis of ITT derivatives, ITT-001 to 006

Melting points were measured with a Yanaco MP-500P apparatus (Yanaco, Kyoto, Japan) and are uncorrected. IR spectra were obtained using a Shimadzu IRPrestige-21 (Shimadzu, Kyoto, Japan). ^1^H- and ^13^C-NMR spectra were obtained on a Bruker AV III 400 (^1^H-NMR: 400 MHz, ^13^C-NMR: 100 MHz) (Bruker Biospin, Rheinstetten, Germany) or JEOL ECA-600II (^1^H-NMR: 600 MHz, ^13^C-NMR: 150 MHz) (JEOL, Tokyo, Japan) instrument for solutions in CDCl_3_, and chemical shifts are reported on the δ scale using TMS as an internal standard of 0.00 for ^1^H-NMR spectra and CDCl_3_ as an internal standard of δ 77.00 for ^13^C-NMR spectra, respectively. MS spectra were measured with a JMS-T100LP (ESI) spectrometer (JEOL).

#### 1-(3,7-Bis(diethylamino)-10*H*-phenoxazin-10-yl)ethanone (ITT-001)

To a solution of basic blue 3 (107.0 mg, 0.30 mmol) in CHCl_3_ (5.4 mL) and H_2_O (5.4 mL) were added 1.05 M NaOH (4.1 mL) and Na_2_S_2_O_4_ (683.0 mg, 1.9 mmol) at ambient temperature. After stirring for 1.5 h at the same temperature, the reaction mixture was added a solution of acetyl chloride (237.0 mg, 3.0 mmol) in CHCl_3_ (8.0 mL). After stirring for 3 h at the same temperature, the reaction mixture was quenched with H_2_O, and extracted with AcOEt. The organic layers were washed with brine, and dried over Na_2_SO_4_. The volatile solvent was removed in vacuo. The residue was purified by column chromatography on silica gel using hexane-AcOEt (95:5, v/v) as eluent to give ITT-001 (85.2 mg, 78%) as a green solid; Mp 148.9–150.8 °C; IR (KBr) ν max 2972, 1661 and 1507 cm^−1^; ^1^H-NMR (CDCl_3_; 400 MHz) δ 7.26 (2H, br s), 6.44–6.33 (4H, br m), 3.32 (8H, q, *J* = 7.0 Hz), 2.29 (3H, s), 1.15 (12H, t, *J* = 7.0 Hz); 13C-NMR δ 169.6, 152.0 (2), 146.7 (2), 125.2 (2), 118.2 (2), 106.1 (2), 99.6 (2), 44.5 (4), 22.8, 12.48 (4); HRMS (ESI-TOF) *m/z*: [M + Na]^+^ Calcd for C_22_H_29_N_3_NaO_2_ 390.2158; Found 390.2163 (Additional file 1: Fig. S1).

#### 1-(3,7-Bis(diethylamino)-10*H*-phenoxazin-10-yl)-2,2-dimethylpropan-1-one (ITT-002)

Reduction of basic blue 3 (300.0 mg, 0.80 mmol), followed by acylation of the resulting phenoxazine with pivaloyl chloride (503.0 mg, 4.2 mmol) was carried out according to the same procedure as described for ITT-001 to give acylphenoxazine ITT-002 (313.8 mg, 90%) as a green solid. [eluent: hexane-AcOEt (9:1, v/v)]; Mp 96.1–97.3 °C; IR (KBr) ν max 2966, 1652, 1507 cm^−1^; ^1^H-NMR (CDCl_3_; 400 MHz) δ 7.35 (2H, d, *J* = 8.8 Hz), 6.42 (2H, d, *J* = 2.6 Hz), 6.35 (2H, dd, *J* = 8.8, 2.6 Hz), 3.31 (8H, q, *J* = 7.0 Hz), 1.20 (9H, s), 1.14 (12H, t, *J* = 7.0 Hz); ^13^C-NMR (CDCl_3_; 100 MHz) δ 178.8, 153.5 (2), 146.7 (2), 125.8 (2), 121.0 (2), 105.8 (2), 99.6 (2), 44.4 (4), 41.3, 29.2 (3), 12.4 (4); HRMS (ESI-TOF) *m/z*: [M + Na]^+^ Calcd for C_25_H_35_N_3_NaO_2_ 432.2627; Found 432.2627 (Additional file 1: Fig. S2).

#### 3,7-Bis(diethylamino)-10*H*-phenoxazin-10-yl)(cyclopropyl)methanone (ITT-003)

Reduction of basic blue 3 (300.0 mg, 0.80 mmol), followed by acylation of the resulting phenoxazine with cyclopropanoyl chloride (436.0 mg, 4.2 mmol) was carried out according to the same procedure as described for ITT-001 to give acylphenoxazine ITT-003 (313.8 mg, 90%) as a gray solid. [eluent: hexane-AcOEt (85:15, v/v)]; Mp 37.7–39.5 °C; IR (KBr) ν max 2969, 1659, 1507 cm^−1^; ^1^H-NMR (CDCl_3_; 400 MHz) δ 7.42 (2H, d, *J* = 9.0 Hz), 6.40 (2H, d, *J* = 2.8 Hz), 6.37 (2H, dd, *J* = 9.0, 2.8 Hz), 3.32 (8H, q, *J* = 7.0 Hz), 2.21-2.09 (1H, m), 1.15 (12H, t, *J* = 7.0 Hz), 1.18-1.13 (2H, m), 0.84-0.77 (2H, m); ^13^C-NMR (CDCl_3_; 100 MHz) δ 172.1, 151.8 (2) 146.4 (2), 125.1 (2), 118.2 (2), 106.0 (2), 99.5 (2), 44.4 (4), 12.4, 12.3 (4), 9.1 (2); HRMS (ESI-TOF) *m/z*: [M + Na]^+^ Calcd for C_24_H_31_N_3_NaO_2_ 416.2314; Found 416.2342 (Additional file 1: Fig. S3).

#### (3,7-Bis(diethylamino)-10*H*-phenoxazin-10-yl)(phenyl)methanone (ITT-004)

Reduction of basic blue 3 (300.0 mg, 0.80 mmol), followed by acylation of the resulting phenoxazine with benzoyl chloride (587.0 mg, 4.2 mmol) was carried out according to the same procedure as described for ITT-001 to give acylphenoxazine ITT-004 (297.6 mg, 83%) as a turquoise solid. [eluent: hexane-AcOEt (80:20, v/v)]; Mp 126.3–127.2 °C; IR (KBr) ν max 2966, 1644, 1507, 1340, 1288, 1241, 1203, 1115 cm^−1^; ^1^H-NMR (CDCl_3_; 400 MHz) δ 7.35 (2H, d, *J* = 7.0 Hz), 7.26-7.17 (3H, m), 7.05 (2H, br d, *J* = 8.0 Hz), 6.33 (2H, d, *J* = 2.5 Hz), 6.11 (2H, dd, *J* = 8.0, 2.5 Hz), 3.21 (8H, q, *J* = 7.0 Hz), 1.05 (12H, t, *J* = 7.0 Hz); ^13^C-NMR (CDCl_3_; 100 MHz) δ 167.4, 151.3 (2), 146.4 (2), 136.1, 129.7, 128.8 (2), 127.8 (2), 125.0 (2), 118.7 (2) 106.1 (2), 99.5 (2), 44.5 (4), 12.4 (4); HRMS (ESI-TOF) *m/z*: [M + H]^+^ Calcd for C_27_H_32_N_3_O_2_ 430.2495; Found 430.2524 (Additional file 1: Fig. S4).

#### 1-(3,7-Bis(diethylamino)-10*H*-phenoxazin-10-yl)-2-(diethylamino)ethanone hydrochloride (ITT-005·HCl)

To a solution of basic blue 3 (3005 mg, 0.8 mmol) in CHCl_3_ (16.0 mL) and H_2_O (16.0 mL) were added 1.0 M NaOH (12.5 mL) and Na_2_S_2_O_4_ (739.0 mg, 4.2 mmol) at ambient temperature. After stirring for 1.5 h at the same temperature, the reaction mixture was added a solution of chloroacetyl chloride (483.0 mg, 4.3 mmol) in CHCl_3_ (24.0 mL). After stirring for 4 h at the same temperature, the reaction mixture was quenched with H_2_O, and extracted with AcOEt. The organic layers were washed with brine, and dried over Na_2_SO_4_. The volatile solvent was removed in vacuo, and the residue was used for the next step without further purification. To a solution of the obtained chloroacetylphenoxazine in THF (2 mL) was added Et_2_NH (186.0 mg, 2.5 mmol) and Bu_4_NI (31.0 mg, 82.0 μmol) at ambient temperature. After stirring for 16 h at 50 °C, the reaction was quenched with 2 M HCl. The aqueous layers were washed with AcOEt, and added 2 M NaOH to extract with AcOEt. The organic layers were washed with brine, dried over Na_2_SO_4_, and concentrated in vacuo. The residue was purified by column chromatography on silica gel using CHCl_3_-MeOH (9005, v/v) as an eluent to give ITT-005 as a turquoise solid. To the solid was added MeOH–HCl, then concentrated in vacuo to give ITT-005·HCl (365.2 mg, 92%) as a yellowish green solid.; Mp 117.5–119.7 °C; IR ν max 3435, 2676, 2597, 1685, 1503 cm^−1^; ^1^H-NMR (CD_3_OD; 400 MHz) δ 7.35 (2H, br s), 6.54-6.45 (4H, m), 3.41 (8H, q, *J* = 7.0 Hz), 3.11 (4H, q, *J* = 7.3 Hz), 1.23 (6H, t, *J* = 7.3 Hz), 1.18 (12H, t, *J* = 7.0 Hz); ^13^C-NMR (CD_3_OD; 100 MHz) δ 167.6, 154.5 (2), 149.9 (2), 126.9 (2), 108.5 (2), 1006 (2), 55.9, 51.5 (2), 46.5 (4), 13.8 (4), 11.2 (2).; HRMS (ESI-TOF) *m/z*: [M + H]^+^ Calcd for C_26_H_39_N_4_O_2_ 439.3073; Found 439.3102 (Additional file 1: Fig. S5).

#### (3,7-Bis(diethylamino)-10*H*-phenoxazin-10-yl)(4-fluorophenyl)methanone (ITT-006)

Reduction of basic blue 3 (302.0 mg, 0.84 mmol), followed by acylation of the resulting phenoxazine with 4-fluorobenzoyl chloride (293.0 mg, 4.2 mmol) was carried out according to the same procedure as described for ITT-001 to give acylphenoxazine ITT-006 (286.8 mg, 77%) as a green solid. [eluent: hexane-AcOEt (75:25, v/v)]; Mp 148.9–150.8 °C; IR (KBr) ν max 2968, 1664, 1508 cm^−1^; ^1^H-NMR (CDCl_3_; 400 MHz) δ 7.42 (2H, d, *J* = 8.8 Hz), 7.11 (2H, d, *J* = 8.6 Hz), 6.94 (2H, d, *J* = 8.8 Hz), 6.41 (2H, d, *J* = 2.8 Hz), 6.21 (2H, dd, *J* = 8.6, 2.8 Hz), 3.31 (8H, q, *J* = 7.0 Hz), 1.38 (12H, t, *J* = 7.0 Hz); ^13^C-NMR (CDCl_3_; 100 MHz) δ 166.3, 151.3 (2), 146.5 (2), 132.7, 131.2 (2), 125.0 (2), 118.6 (2), 115.6, 114.9 (2), 106.2 (2), 99.6 (2), 44.5 (4) 12.4 (4); HRMS (ESI-TOF) *m/z*: [M + H]^+^ Calcd for C_27_H_31_FN_3_O_2_ 448.2400; Found 448.2422 (Additional file 1: Fig. S6).

### Compounds

Basic blue 3 and ITT-001 to 006 were synthesized as described above. ART and chloroquine (CQ) were purchased from TCI (Tokyo, Japan). CQ was dissolved in saline, while all other chemicals were dissolved in dimethyl sulfoxide (DMSO) (Nacalai Tesque, Kyoto, Japan) to obtain 10 mM stock solution. All solutions were stored at − 30 °C and thawed immediately prior to experimentation. The stock solutions were subjected to a maximum of five freeze–thaw cycles before being discarded.

### Parasite strains and culture

*Plasmodium falciparum* laboratory strains, 3D7 [CQ- and pyrimethamine (PYR)-sensitive] and W2 (CQ- and PYR-resistant) were obtained from Prof. Masatsugu Kimura (Osaka City University, Osaka, Japan) and the Malaria Research and Reference Reagent Resource (MR4) as part of the Biodefense and Emerging Infections (BEI) Resources, respectively. The field isolates, MRA-1239 and MRA-1240 (ART-sensitive and ART-resistant, respectively), were obtained from MR4, BEI Resources. For the assessment of anti-malarial activity of the compounds in vitro, the parasites were cultured in Roswell Park Memorial institute (RPMI) 1640 medium supplemented with 0.5 g/L l-glutamine, 5.96 g/L HEPES, 2 g/L sodium bicarbonate (NaHCO_3_), 50 mg/L hypoxanthine, 10 mg/L gentamicin, 10% heat-inactivated human serum, and red blood cells (RBCs) at a 3% haematocrit in an atmosphere of 5% CO_2_, 5% O_2_, and 90% N_2_ at 37 °C as previously described [25]. RBCs infected with ring-form were collected using the sorbitol synchronization technique [26]. Briefly, the RBCs were collected by centrifugation at 840*g* for 5 min at room temperature, suspended in a fivefold volume of 5% d-sorbitol (Nacalai Tesque) for 10 min at room temperature, and then washed twice with RPMI 1640 medium to remove the d-sorbitol.

### Characterization of anti-malarial activity in vitro

The ring-form-synchronized parasite (3D7 strain) was treated with the indicated concentrations of basic blue 3 and ITT-001 to 006 for 24 h for flow cytometric and morphological analyses using an automated haematology analyzer, XN-30, and light microscope, respectively.

### The automated haematology analyzer, XN-30

For the flow cytometric analysis, the XN30 analyzer was equipped with a prototype algorithm for cultured *P. falciparum* [prototype; software version: 01-03, (build 16)] and specific reagents were used (CELLPACK DCL, SULFOLYSER, Lysercell M, and Fluorocell M) (Sysmex, Kobe, Japan) [23]. Approximately 100 µL of the culture suspension diluted with 100 µL phosphate-buffered saline was added into a BD Microtainer MAP Microtube for Automated Process K_2_ EDTA 1.0 mg tube (Becton–Dickinson and Co., Franklin Lakes, NJ, USA) and loaded onto the XN-30 analyzer with an auto-sampler as described in the instrument’s manual (Sysmex) [24]. The XN-30 analyzer provided the M scattergram, which showed developmental stages of parasites (see Fig. 2, DMSO) according to DNA content and parasite-infected RBC (iRBC) size [23]. Horizontal and vertical axes represent the intensities of side fluorescent light (SFL, which corresponds to DNA content) and forward scattered light (FSC, indicating iRBC size), respectively. Colours indicate the following based on the default setting: red, ring-form; orange, trophozoite; purple, schizont; and blue, polychromatic RBC. Once parasites replicate DNA, dots are plotted to the right side on the M scattergram according to the increase in DNA content [23]. The parasitaemias (total, MI-RBC%; ring-form, RNG-RBC%; trophozoite, TRPZ-RBC%; and schizont, SCHZ-RBC%) were automatically reported.

### Microscopy

A standard thin blood smear was fixed with 100% methanol for 10 min and stained with 10% Giemsa working solution, pH 7.2 (Merck KGaA, Darmstadt, Germany) for 13 min. Slides were observed at 1000× magnification using the model BX50 light microscope (Olympus, Tokyo, Japan).

### Measurement of anti-malarial efficacy using the XN-30 analyzer

Ring-form-synchronized parasites were cultured with basic blue 3, ITT-001 to 006, CQ, and ART at sequentially decreasing concentrations (5, 1.5, 0.5, 0.15, 0.05 0.015, 0.005, and 0.0015 μM) for 48 h. The parasitaemia was measured with the XN-30 analyzer as mentioned above. DMSO (0.5%) alone or containing 5 µM ART was used as the negative or positive control, respectively. The growth inhibition (GI) rate was calculated from the MI-RBC% according to the following equation:

$${\text{GI }}(\% )\, = \, 100\, - \,({\text{test sample}}\, - \,{\text{positive control}})/({\text{negative control}}\, - \,{\text{positive control}})\, \times \, 100.$$

The GI [%, mean ± standard error of the mean (SEM)] was calculated using the Microsoft Excel program (Microsoft, Redmond, WA, USA). The data were graphically displayed and IC_50_ was calculated using GraphPad Prism version 5.0 (GraphPad Prism Software, San Diego, CA, USA) [25].

### Measurement of fluorescence spectrum

Fluorescence spectrum of basic blue 3 and ITT-001 to 006 (10 µM) was obtained in saline containing 1% DMSO at room temperature. The compounds were excited at 592 nm (bandpass width, 5 nm) and their emission spectra, 630 nm to 730 nm, were measured with a RF-5300PC Spectro fluorophotometer (Shimadzu).

### Fluorescence microscopy

Non-synchronized parasites were washed with Hanks’ balanced salt solution (HBSS) containing calcium and magnesium (Thermo Fisher Scientific, Waltham, MA, USA) twice by centrifugation at 840*g* for 5 min at room temperature and treated with 15 nM basic blue 3 in HBSS containing calcium and magnesium for 15 min under standard culture conditions. Simultaneously, DNA was stained with 50 µg/mL Hoechst 33342 (Dojindo) for 5 min. The parasites were washed with HBSS containing calcium and magnesium twice. Images were captured using a BZ-X710 fluorescence microscope (Keyence, Osaka, Japan). A BZ-X TexasRed filter (OP-87765; excitation = 560/40 nm, emission = 630/75 nm, and dichroic mirror = 585 nm) was used for basic blue 3.

### Cytotoxic assay

HepG2 (JCRB1054) and HEK293T cell lines were obtained from the Japanese Collection of Research Bioresources (JCRB, Osaka, Japan) and Prof. Yoshiharu Matsuura (Osaka University, Osaka, Japan) [27], respectively. The cells were cultured in Dulbecco’s modified Eagle’s medium (DMEM (1.0 g/L glucose) with l-glutamine and sodium pyruvate; Nacalai Tesque, Kyoto, Japan) supplemented with 10% (v/v) fetal bovine serum (FBS; Gibco-BRL, Grand Island, NY, USA) in a humidified incubator with 5% CO_2_ at 37 °C. For the cytotoxic assay, the cells (5 × 10^3^/well) were seeded in a 96-well plate. Basic blue 3, ITT-001 to 006, and CQ at gradually decreasing concentrations (50, 15, 5, 1.5, 0.5, 0.15, 0.05, and 0.015 μM) were added to the cell culture after 24 h and the cells were subsequently cultured for 48 h. Cell viability was measured using a Cell Counting Kit-8 (Dojindo) according to the manufacturer’s instructions. Briefly, 10 µL CCK-8 reagent was added to each well containing culture medium and incubated for 2 h under standard culture conditions. The absorbance of the sample was measured at 450 nm using a PowerWave HT microplate spectrophotometer (BioTek Instruments, Winooski, VT, USA). The cell viability was expressed as a percentage of the absorbance of the untreated control cells after subtracting the appropriate background intensity. The CC_50_ (mean ± SEM) was calculated as described in IC_50_. The SI, which is the ratio between the cytotoxicity and anti-malarial activity, was calculated according to the following equation.

$${\text{SI}}\, = \,{\text{mean CC}}_{ 50} /{\text{mean IC}}_{ 50}.$$

### In vivo anti-malarial activity

The in vivo anti-malarial efficacy of ITT-001 to 006 was evaluated using the rodent malaria parasite *P. berghei* NK65 as described by Fidock et al. [28], with some modifications. The parasite was maintained by syringe passage every week in female ICR mice (4–8-week-old; body weight, 25–27 g, Sankyo Lab Service, Tokyo, Japan). On day 0, a 200 µL aliquot of saline containing 2 × 10^7^ iRBCs prepared from the pre-infected mice was injected intravenously into the mice. After 4 h, basic blue 3 and ITT-001 to 006 dissolved or suspended in 5% (w/v) methyl cellulose 400 solution (FUJIFILM Wako Pure Chemical, Osaka, Japan) were orally administered (100 mg/kg body weight of basic blue 3 and ITT-001 to 006 and 50 mg/kg body weight of ITT-004) to the mice (four and five groups for basic blue 3 and ITT-001 to 006, respectively). The control group (five mice/group) was treated with approximately 100 µL of the vehicle. This treatment was repeated for 3 consecutive days. On day 4, peripheral blood samples were obtained from the tail veins of the mice and thin blood smears stained with Diff-Quik stain kit (Sysmex) were observed under a Leica DM6000B microscope (Leica Microsystems, Wetzlar, Germany). Parasitaemia was determined by counting over 10 fields of view. The suppression rate (%) was calculated as the difference in parasitaemia between the test and control groups. The mean survival day (MSD) was calculated from the number of the surviving mice on day 30. The surviving mice were cured on day 30. Kaplan–Meier curves were generated to graphically display the efficacy using GraphPad Prism version 5.0.

### Statistical analysis

The statistical significance was calculated using a two-tailed unpaired Student’s *t* test. The significance limit was set at P < 0.05 and calculations were performed using the GraphPad Prism version 5.0.

## Results

### Synthesis of ITT derivatives

**Fig. 1:**
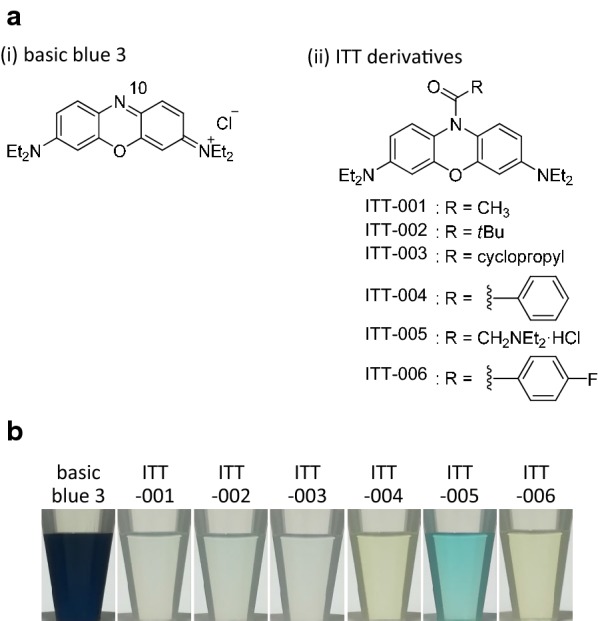
Synthesis and structure of ITT derivatives. **a** Structure of basic blue 3 (i) and ITT derivatives (ITT-001 to 006) (ii). (i) The “10” indicates the nitrogen atom at the 10-position of the phenoxazine ring, which was modified to acyl group as shown in (ii). (ii) “R” indicates alkyl group replaced with an acyl group as shown in lower panel. **b** Visual confirmation of colour of basic blue 3 and ITT-001 to 006. The compounds were visualized at 1 mM in dimethyl sulfoxide (DMSO)

To characterize the effects of the modification of the nitrogen atom at the 10-position on the phenoxazine ring of basic blue 3 [Fig. 1a(i)], six derivatives modified with a variety of acyl groups were synthesized [Fig. 1a(ii), ITT-001 to 006]. The comparison of their colours showed that ITT-001 to 003 and 005 were light blue, and ITT-004 and 006 were light yellow (Fig. 1b), indicating that the modification resulted in fading.

### Anti-malarial efficacy of ITT derivatives in vitro

For both 3D7 (CQ- and PYR-sensitive) and W2 (CQ- and PYR-resistant) strains, the in vitro anti-malarial assay revealed that the efficacy of ITT-001 to 004 and 006 (IC_50_ = approximately 10 nM) was lower than that of basic blue 3 (IC_50_ = approximately 4 nM); the IC_50_ of ITT-005 (about 40 nM) was approximately tenfold higher than that of basic blue 3. The efficacy of these compounds was comparable against 3D7 and W2 strains, although CQ significantly affected the 3D7 strain more than the W2 strain (Table 1). The anti-malarial activity of basic blue 3 and ITT-001 to 006 was almost the same against MRA-1239 (ART-sensitive) and MRA-1240 (ART-resistant) isolates, whereas that of CQ and ART against MRA-1240 isolate was weaker than that against MRA-1239 isolate (Table 1). These results suggested that these compounds were effective on CQ- and PYR-resistant strains and ART-resistant field isolates as well as the sensitive ones.

**Table 1:** Anti-malarial activity of basic blue 3 and ITT derivatives in vitro

	3D7	W2	MRA-1239	MRA-1240
	IC_50_ (nM)^a^	IC_50_ (nM)^a^	IC_50_ (nM)^a^	IC_50_ (nM)^a^
basic blue 3	3.2 ± 0.19	4.8 ± 0.53	5.2 ± 1.1	5.9 ± 0.68
ITT-001	11.2 ± 1.3	11.1 ± 1.8	34.6 ± 9.1	39.0 ± 11.2
ITT-002	10.7 ± 1.5	10.4 ± 2.8	20.7 ± 1.6	24.4 ± 3.7
ITT-003	9.8 ± 1.0	9.6 ± 2.1	24.9 ± 3.1	31.3 ± 9.8
ITT-004	11.3 ± 2.4	12.3 ± 2.7	26.4 ± 2.9	30.5 ± 2.0
ITT-005	138.3 ± 9.0	145.6 ± 29.5	130.2 ± 9.3	183.7 ± 30.1
ITT-006	10.6 ± 1.6	16.0 ± 4.5	27.5 ± 3.4	29.4 ± 5.8
CQ	8.6 ± 0.21	225.0 ± 23.2***	38.6 ± 5.2	58.0 ± 2.6*
ART	ND	ND	5.5 ± 0.018	13.2 ± 1.4**

*IC*_*50*_ concentration of 50% inhibition of parasite growth, *SEM* standard error of the mean, *CQ* chloroquine, *ART* artemisinin, *ND* not determine

IC_50s_ were compared between 3D7 and W2 strains and between MRA-1239 and MRA-1240 isolates (two-tailed unpaired Student’s *t*-test: * P < 0.05; ** P < 0.01; and *** P < 0.001)

^a^Data represent the mean ± SEM values for three experiments

**Fig. 2:**
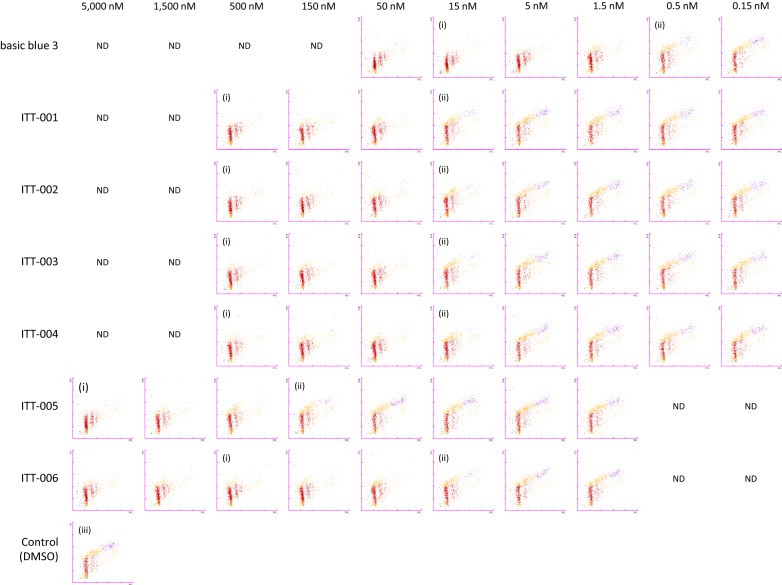
Flow cytometric analysis of anti-malarial activity of basic blue 3 and ITT derivatives in vitro. Representative M scattergrams where “(i)”, “(ii)”, and “(iii)” indicate samples in which parasite morphology was captured (as shown in Fig. 3), ND, not determined; red, ring-form; orange, trophozoite; purple, schizont; and blue, polychromatic red blood cell (RBC) markers were assigned based on default setting of the XN-30 analyzer. Horizontal and vertical axes indicate side fluorescent light (SFL, corresponding to DNA content) and forward scattered light (FSC, indicating size of infected RBCs), respectively [25, 35]. This assay was conducted using 3D7 strain

**Fig. 3:**
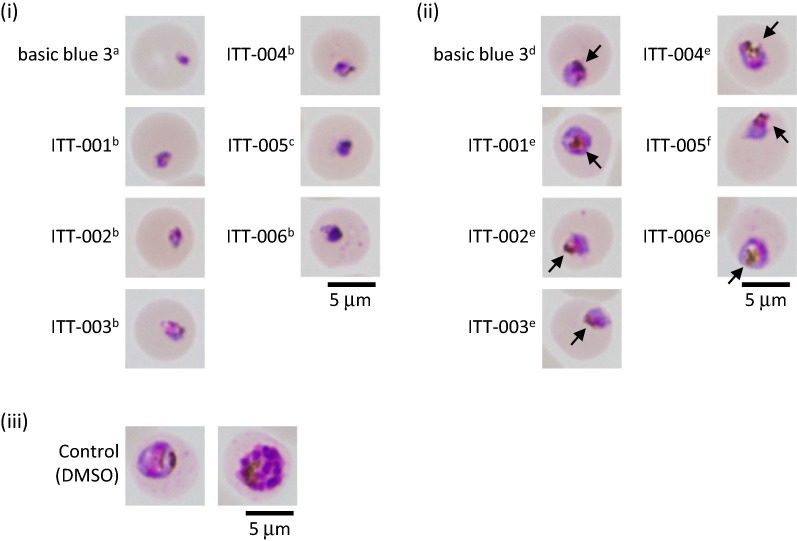
Morphological analysis of anti-malarial activity of basic blue 3 and ITT derivatives in vitro. Representative light microscopy images captured at indicated concentrations “(i)”, “(ii)”, and “(iii)” in Fig. 2. (i) Letters “a”, “b”, and “c” denote final concentrations of 15, 500, and 5000 nM, respectively. (ii) Letters “d”, “e”, and “f” denote final concentrations of 0.5, 15, and 150 nM, respectively. Arrow indicates haemozoin. (iii) Control treated with solvent (DMSO, no compounds). This assay was conducted with 3D7 strain. Scale bar, 5 µm

To further characterize the efficacy of ITT-001 to 006 on parasites in vitro, the 3D7 strain was treated with basic blue 3 and ITT-001 to 006 for 24 h. Analysis with the XN-30 analyzer showed a Type I outcome [24]; that is, parasites were arrested or killed with the ring-form after treatment with compounds at 15 nM (basic blue 3), 500 nM (ITT-001 to 004 and 006), or 5000 nM (ITT-005, Fig. 2i). Trophozoites appeared at 0.5 nM basic blue 3, 15 nM ITT-001 to 004 and 006, and 150 nM ITT-005 (Fig. 2ii), whereas most parasites treated with DMSO developed to trophozoites and schizonts (Fig. 2iii). These results indicated that basic blue 3 showed the strongest anti-malarial activity, followed by ITT-001 to 004 and 006, while the weakest was ITT-005, which was similar to the outcome of treatment for 48 h (Table 1). In addition, the results obtained using the XN-30 analyzer indicated that no compounds disrupted RBCs for 24 h. The light microscopy on Giemsa-stained slides revealed that parasites shrank and appeared to have been killed by ITT-001 to 006, consistent with the effects of basic blue 3. Furthermore, these compounds acted before haemozoin formation (Fig. 3i). However, parasites synthesized haemozoin at suboptimal concentrations, suggesting that the primary efficacy of these compounds was not inhibition of haemozoin formation (Fig. 3ii, arrows).

### Localization of basic blue 3 in vitro

**Fig. 4:**
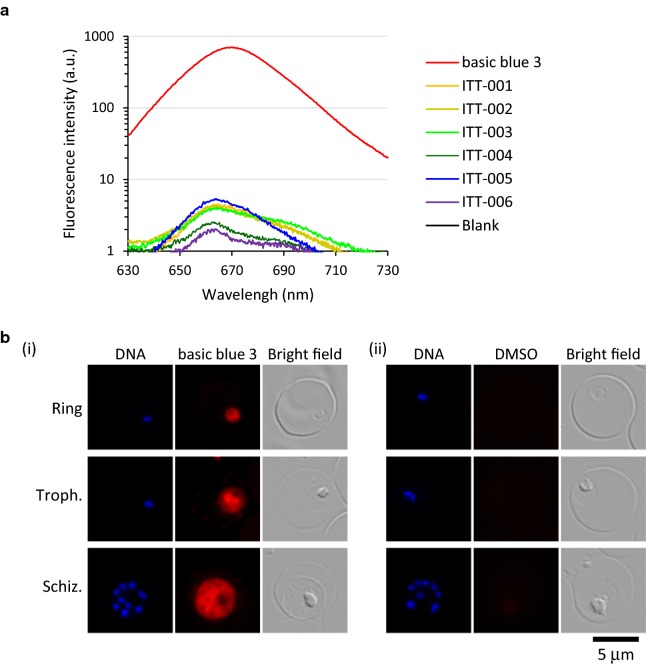
Localization of basic blue 3 in vitro. **a** Representative data of self-fluorescence intensity of basic blue 3 and ITT-001 to 006. Red, basic blue 3; orange, ITT-001; dark yellow, ITT-002; light green, ITT-003; dark green, ITT-004; blue, ITT-005; purple, ITT-006; and black, DMSO, a.u., arbitrary unit. **b** Representative images of live parasite (3D7 strain) treated with 15 nM basic blue 3 (i) and DMSO (ii). Ring, ring-form; Troph., trophozoite; and Schiz., schizont. Red and blue indicate basic blue 3 and DNA, respectively. Scale bar, 5 µm

Benzo[*a*]phenoxazine has self-fluorescence activity [22], implying that basic blue 3 and ITT-001 to 006 with the phenoxazine ring would also have self-fluorescence activity. The measurement showed that the maximum fluorescence intensity of basic blue 3 was 775 at 667 nm, whereas that of ITT-001 to 006 was less than 5. Especially, the intensity of benzamides ITT-004 and 006 was less than 3. The activity of each derivative was > 150-fold weaker than that of basic blue 3 (Fig. 4a). This finding suggested that the acylation of the amino group at the 10-position on the phenoxazine ring disrupted the self-fluorescence activity. Fluorescence microscopy revealed that basic blue 3 was localized to the plasmodial cytoplasm in the ring-form, trophozoite, and schizont stages (Fig. 4b). In contrast, ITT-001 to 006 that had extremely weak signals, as shown in Fig. 4a, were undetectable. Although ITT-001 to 006 were not detectable, their anti-malarial efficacy suggests that they are also selectively accumulated in the plasmodial cytoplasm and play a key role in inhibiting parasite growth.

### Cytotoxicity of ITT derivatives in vitro

Evaluation of the cytotoxicity of the test compounds revealed that basic blue 3 had the highest cytotoxicity (the half-maximal (50%) cytotoxic concentration (CC_50_) = 0.021 and 1.0 µM for HEK293T and HepG2 cells, respectively), whereas ITT-003, 004, and 006 had the lowest cytotoxicity (Table 2: CC_50_ = 9.2, 10.5, and 13.6 µM for HEK293T cells and each > 50 µM for HepG2 cells). The selective index (SI) indicated that ITT-003, 004, and 006 showed the highest effectiveness on the parasites and were the safest for both cultured cells in vitro (Table 2: SI = each > 290 for HEK293T cells and > 1500 for HepG2 cells).

**Table 2:** Cytotoxicity and selective index (SI) of basic blue 3 and ITT derivatives in vitro

	HEK293T					HepG2				
	CC_50_ (μM)^a^	SI				CC_50_ (μM)^a^	SI			
		3D7	W2	MRA-1239	MRA-1240		3D7	W2	MRA-1239	MRA-1240
basic blue 3	0.021 ± 0.0042	6.6	4.4	4.0	3.6	1.0 ± 0.064	312.5	208.3	192.3	169.5
ITT-001	0.32 ± 0.042	28.6	28.8	9.2	8.2	4.0 ± 0.85	357.1	357.1	90.1	12.6
ITT-002	0.20 ± 0.021	18.7	19.2	9.7	8.2	> 50	> 4672.9	> 4807.7	> 2415.5	> 2049.2
ITT-003	9.2 ± 3.8	938.8	958.3	369.5	293.9	> 50	> 5102.0	> 5208.3	> 2008.0	> 1597.4
ITT-004	10.5 ± 3.4	929.2	853.7	397.7	344.3	> 50	> 4424.8	> 4065.0	> 1893.9	> 1639.3
ITT-005	0.73 ± 0.093	5.3	5.0	5.6	4.0	4.6 ± 0.26	33.2	31.6	35.3	25.0
ITT-006	13.6 ± 3.0	1283.0	850.0	494.5	462.6	> 50	> 3676.5	> 3125.0	> 1818.2	> 1700.7
CQ	2.9 ± 0.27	337.2	12.9	75.2	50.0	1.8 ± 0.14	209.3	8.0	46.6	31.0

*CC*_*50*_ concentration of 50% cytotoxicity, *SI* selective index, *CQ* chloroquine

^a^Data represent the mean ± SEM values for three experiments

### Anti-malarial activity of ITT derivatives in vivo

**Fig. 5:**
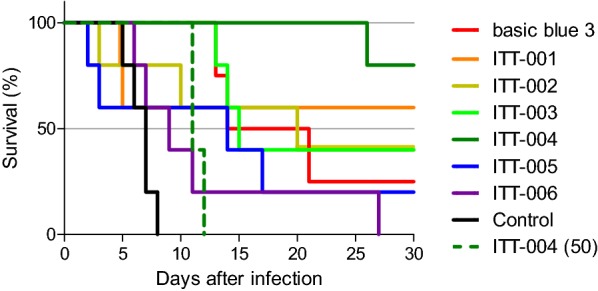
Anti-malarial activity of basic blue 3 and ITT derivatives in vivo. Kaplan–Meier curves of test groups. Solid lines indicate groups administered 100 mg/kg basic blue 3 and ITT-001 to 006 per mouse. Dash line indicates group administered 50 mg/kg ITT-004 per mouse. Red, basic blue 3; orange, ITT-001; dark yellow, ITT-002; light green, ITT-003; dark green, ITT-004; blue, ITT-005; purple, ITT-006; and black, control (Additional file 1: Fig. S7)

The in vivo anti-malarial efficacy of ITT-001 to 006 was evaluated following oral administration to mice infected with the rodent malaria parasite, *P. berghei* NK65. Basic blue 3 and ITT-001 to 006 showed higher anti-malarial efficacy than did the vehicle control treatment (Fig. 5 and Table 3). In addition, these compounds exhibited high suppression rates on day 4 (Table 3). Comparison of the MSD values revealed that all treated groups showed longer survival than that of the control group (Table 3). Especially, ITT-004 showed the highest MSD (29.4 at 100 mg/kg), and four of five mice were considered as cured on day 30 (Fig. 5 and Table 3). These outcomes of ITT-004 were consistent with its highest anti-malarial activity and in vitro selectivity (Tables 1, 2). The administration of half the standard dose (50 mg/kg per mouse) of ITT-004 was also effective compared with the vehicle [Fig. 5, dark green dotted line; and Table 3, ITT-004 (50)].

**Table 3:** Anti-malarial activity of basic blue 3 and ITT derivatives in vivo

	% suppression on day 4	MSD (day)	# of survived mice on day 30
basic blue 3^a^	> 99	20.3	1/4
ITT-001^a^	> 99	20.4	3/5
ITT-002^a^	> 99	19.2	2/5
ITT-003^a^	> 99	21.0	2/5
ITT-004^a^	> 99	29.4	4/5
ITT-005^a^	> 99	14.0	1/5
ITT-006^a^	91.5	13.0	0/5
ITT-004 (50)^b^	> 99	11.8	0/5
Control	–^c^	6.5 ± 0.9^d^	0/5 (per group)

*MSD* mean survival day

^a^Orally administered to mice at a dose of 100 mg/kg

^b^Orally administered to mice at a dose of 50 mg/kg

^c^Parasitaemia: 25.9 ± 5.8%^d^

^d^Mean ± SEM of representative three groups (see also Additional file 1: Fig. S7)

## Discussion

Basic blue 3 is a potent anti-malarial compound, but it has a dark blue colour and relatively high cytotoxicity (Fig. 1b, Tables 1 and 2) [11, 20]. In the current study, six ITT derivatives (ITT-001 to 006) in which the amino group at the 10-position on the phenoxazine ring of basic blue 3 was modified with a variety of acyl groups (Fig. 1a). The colour comparison revealed that acylation of the amino group at the 10-position on the phenoxazine ring obviously affected the colour of the test compounds. Thus, ITT-001 to 003 and 005 were light blue, while ITT-004 and 006 were light yellow (Fig. 1b). Furthermore, fluorometry revealed that the modification decreased the self-fluorescence of basic blue 3 (Fig. 4a). These findings suggested that the acylation of the amino group at the 10-position on the phenoxazine ring determined the colour and intensity of self-fluorescence.

The XN-30 analyzer showed that treatment with high concentrations of basic blue 3 and ITT-001 to 006 either killed the parasites or arrested them before DNA replication (Fig. 2i). In addition, light microscopy revealed haemozoin formation in parasites treated with intermediate concentrations of the compounds (Fig. 3ii, arrows). These findings suggested that these compounds did not primarily inhibit haemozoin formation.

Fluorescence microscopy showed that basic blue 3 was selectively accumulated in the plasmodial cytoplasm (Fig. 4b). In contrast, the localization of ITT-001 to 006 was not detectable because of their low fluorescence activity (Fig. 4a). However, they, especially ITT-003, 004, and 006, exhibited lower cytotoxicity toward HEK293T and HepG2 cells (Table 2), suggesting that they more selectively accumulated in unspecified plasmodial organelles in the cytoplasm and that parasites were killed through any function.

It has been reported that rhodamine dyes, such as rhodamine 123, and rhodacyanin dyes, such as MKT-077, which are members of the DLC, target F0F1 ATPase (Complex V) and prevent ATP synthesis [29]. Both findings, (i) the structures of the basic blue 3 and its derivatives that are similar to that of rhodamine 123 (Fig. 1a) [29] and (ii) the localization of basic blue 3 to the plasmodial cytoplasm containing mitochondria (Fig. 4b), imply that they target the *P. falciparum* mitochondrial Complex V and prevent ATP synthesis of parasites. Previous studies reported that the mitochondrial electron transport chain that contains Complex V is dispensable in the blood stage but essential in the mosquito phase [30–33]. In addition, a recent report showed that the development of *P. falciparum* can be rapidly and completely blocked in female *Anopheles gambiae* mosquitoes treated with specific anti-malarial, atovaquone [34]. These facts provide one possibility of the mechanism of action in their anti-malarial efficacy, but further analyses would be required to elucidate the mechanism.

Compounds ITT-003, 004, and 006 showed lower cytotoxicity and higher selectivity than basic blue 3 did in vitro (Table 2). In contrast, the in vivo assay revealed that ITT-004 was the most effective on *P. berghei* parasite infection, with 4 out of 5 animals cured on day 30 (Fig. 5 and Table 3). Benzoyl groups on both ITT-004 and 006 may be important for low cytotoxicity. These differences likely determined the efficacy in vivo, but further analyses would be required. Although in this study the in vitro and in vivo assays demonstrated that ITT-004 would be a promising anti-malarial lead compound, further analyses on the mechanism of action underlying the parasite killing efficacy of ITT-004 would expand its potential.

Although ITT-004 showed the highest anti-malarial activity in the in vivo rodent malaria model (Fig. 5 and Table 3), its dosage (100 mg/kg) was relatively high. Previously, some basic blue 3 derivatives in which nitrogen atoms are bonded to the carbon atoms at the 3- and 7-positions on the phenoxazine ring showed potent antiprotozoal activity against *P. falciparum*, *Trypanosoma cruzi*, *Trypanosoma brucei rhodesiense*, and *Leishmania donovani* parasites in vitro [20]. A combination of these derivatives with ITT-004 would be valuable for obtaining a higher in vivo anti-malarial activity or lower dosage.

The causative pathogenic parasites of neglected tropical diseases, such as trypanosomiasis and leishmaniasis, belong to the Trypanosomatidae family in the Euglenozoa phylum. It was previously reported that some other derivatives of basic blue 3 showed potent antiprotozoal activity in vitro [20]. Further analysis is required to evaluate the antiparasitic efficacy of ITT-001 to 006, especially ITT-003, 004, and 006, which showed low cytotoxicity.

## Conclusions

The six ITT derivatives were effective against CQ- and PYR-resistant strains and ART-resistant field isolates as well as the sensitive ones. Among them, ITT-004, which had high anti-malarial activity and low cytotoxicity in vitro and in vivo, is a promising anti-malarial lead compound.

## Additional file

**Additional file 1: Fig. S1.** ^1^H- and ^13^C-NMR Spectra of ITT-001. **Fig. S2.** ^1^H- and ^13^C-NMR Spectra of ITT-002. **Fig. S3.** ^1^H- and ^13^C-NMR Spectra of ITT-003. **Fig. S4.** ^1^H- and ^13^C-NMR Spectra of ITT-004. **Fig. S5.** ^1^H- and ^13^C-NMR Spectra of ITT-005·HCl. **Fig. S6.** ^1^H- and ^13^C-NMR Spectra of ITT-006. **Fig. S7.** Kaplan–Meier curve of representative control groups. Group denoted as “#3” is control group (black line) in Fig. 5.

## Data Availability

The datasets used and/or analysed during the current study are available from the corresponding author on reasonable request.

## Abbreviations

WHO: World Health Organization
ART: artemisinin
ACTs: ART-based combination therapies
SEA: Southeast Asia
DLC: π-delocalized lipophilic cations
RBC: red blood cell
iRBC: infected RBC
FSC: forward scattered light
SFL: side fluorescent light
CQ: chloroquine
PYR: pyrimethamine
DMSO: dimethyl sulfoxide
SEM: standard error of the mean
MSD: mean survival day
