# Supplementary material for: In vitro and in vivo characterization of anti-malarial acylphenoxazine derivatives prepared from basic blue 3
Source: Malar J. 2019 Jul 15;18:237. doi: 10.1186/s12936-019-2873-0 (PMC6631887; doi:10.1186/s12936-019-2873-0)
Supplement: Supplementary file 1 — Additional file 1: Fig. S1. 1H- and 13C-NMR Spectra of ITT-001. Fig. S2. 1H- and 13C-NMR Spectra of ITT-002. Fig. S3. 1H- and 13C-NMR Spectra of ITT-003. Fig. S4. 1H- and 13C-NMR Spectra of ITT-004. Fig. S5. 1H- and 13C-NMR Spectra of ITT-005·HCl. Fig. S6. 1H- and 13C-NMR Spectra of ITT-006. Fig. S7. Kaplan–Meier curve of representative control groups. Group denoted as “#3” is control group (black line) in Fig. 5. [file 12936_2019_2873_MOESM1_ESM.docx]

Fig. S1: ^1^H- and ^13^C-NMR Spectra of ITT-001


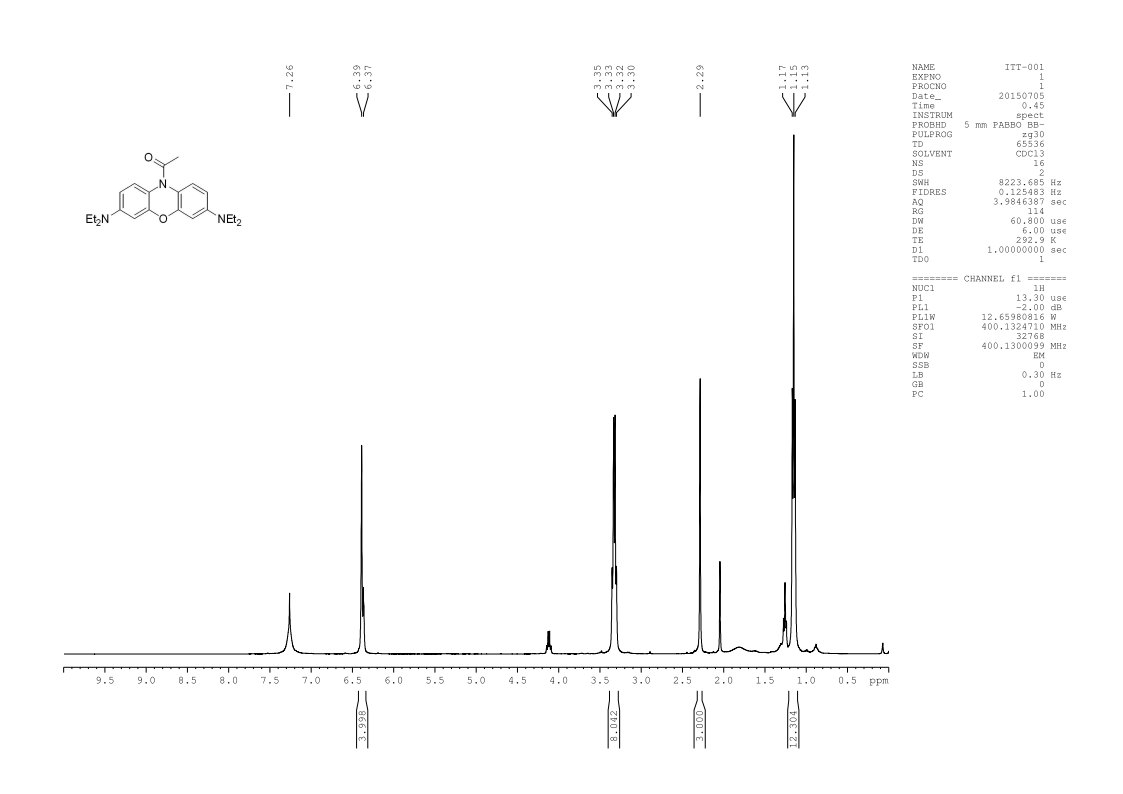


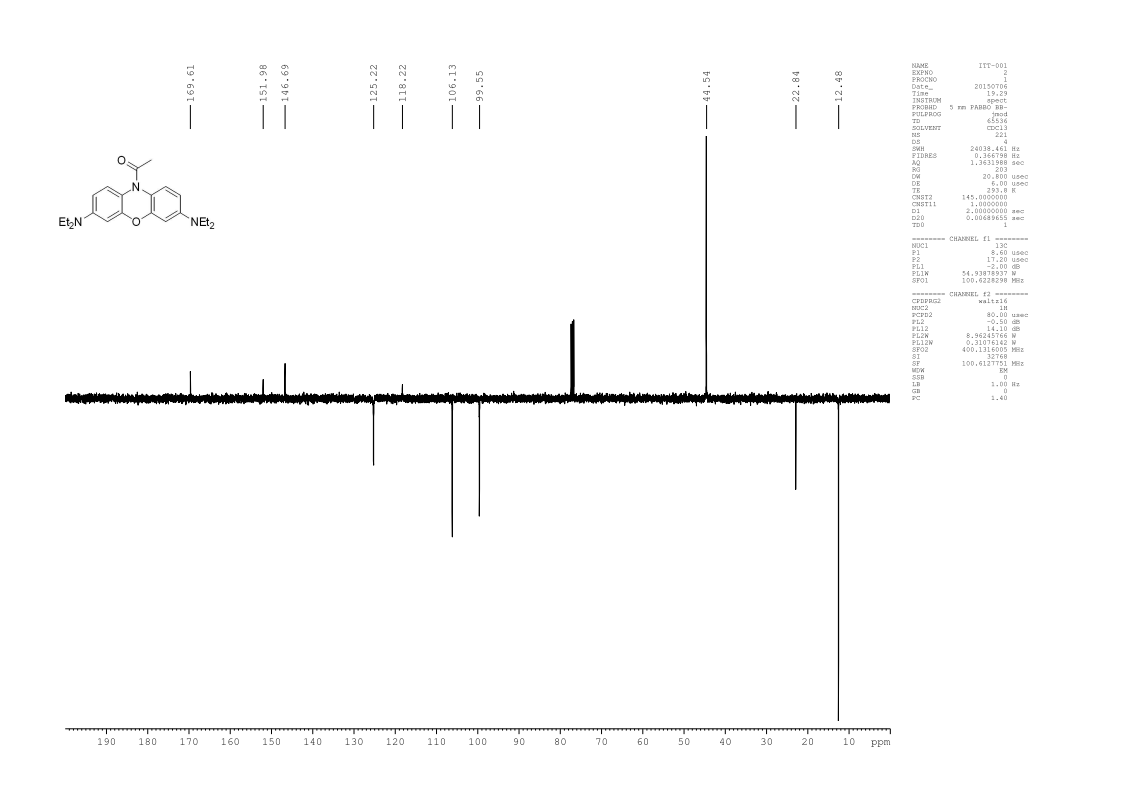


Fig. S2: ^1^H- and ^13^C-NMR Spectra of ITT-002


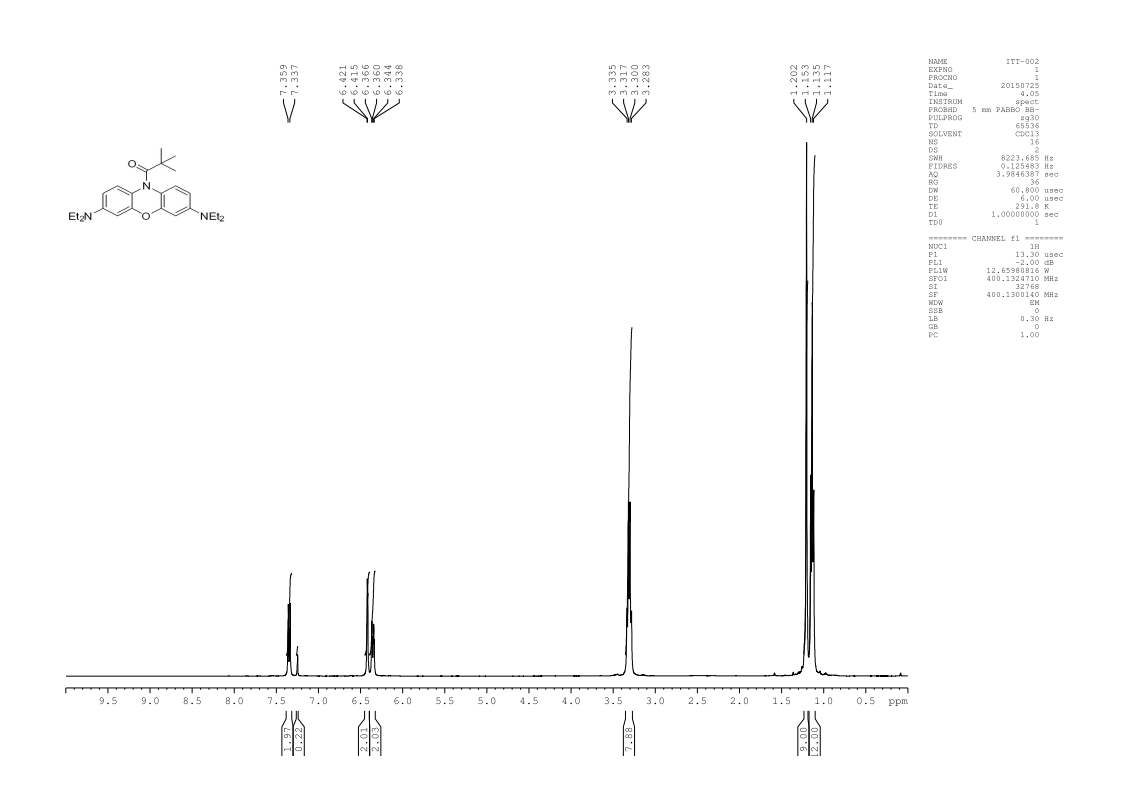

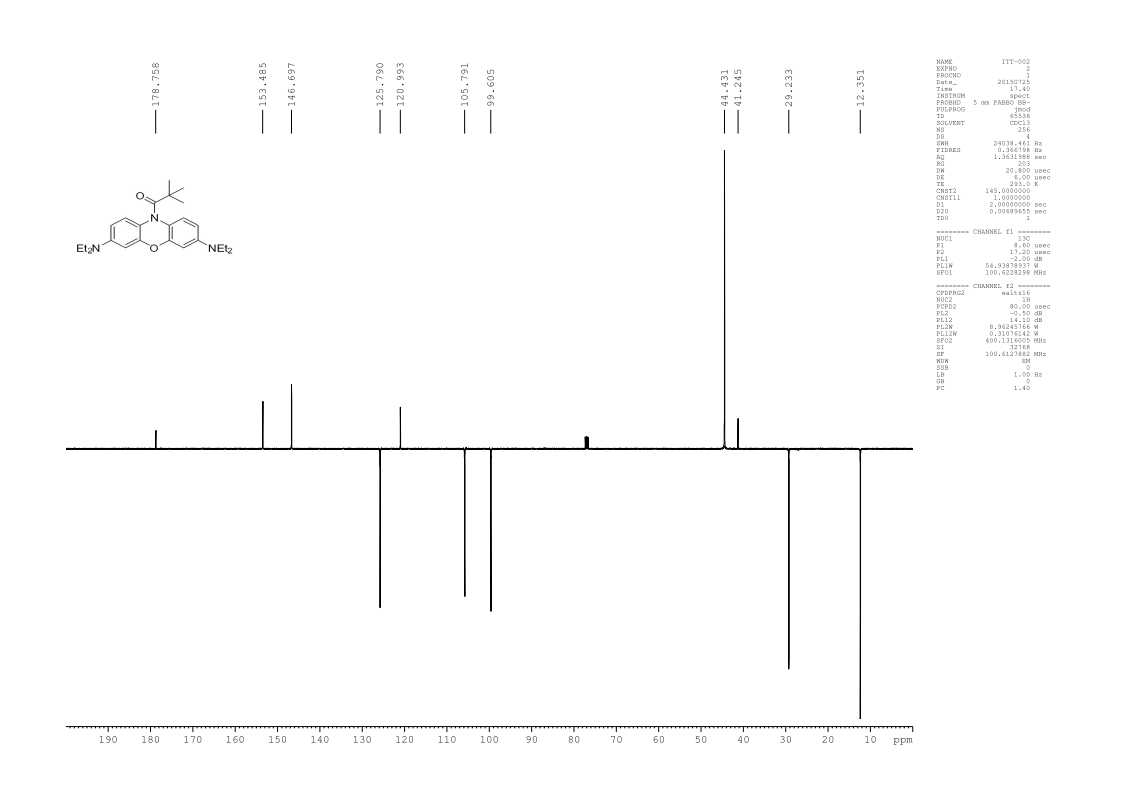


Fig. S3: ^1^H- and ^13^C-NMR Spectra of ITT-003


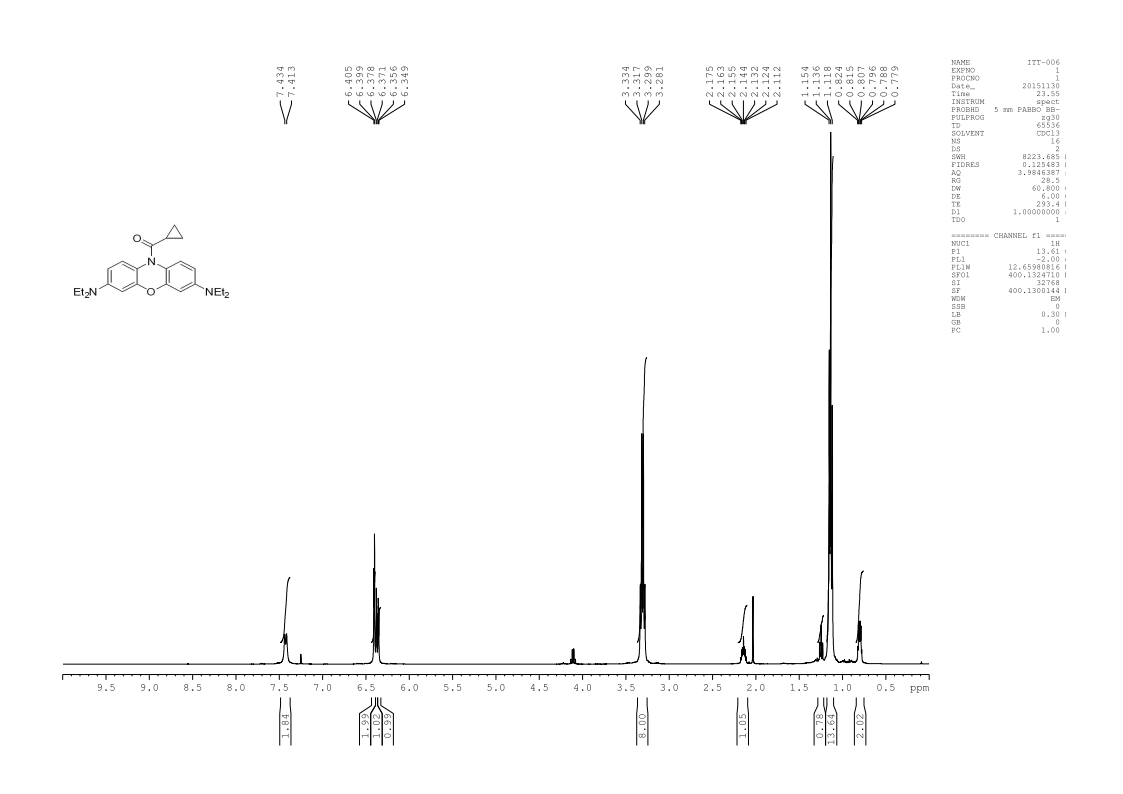

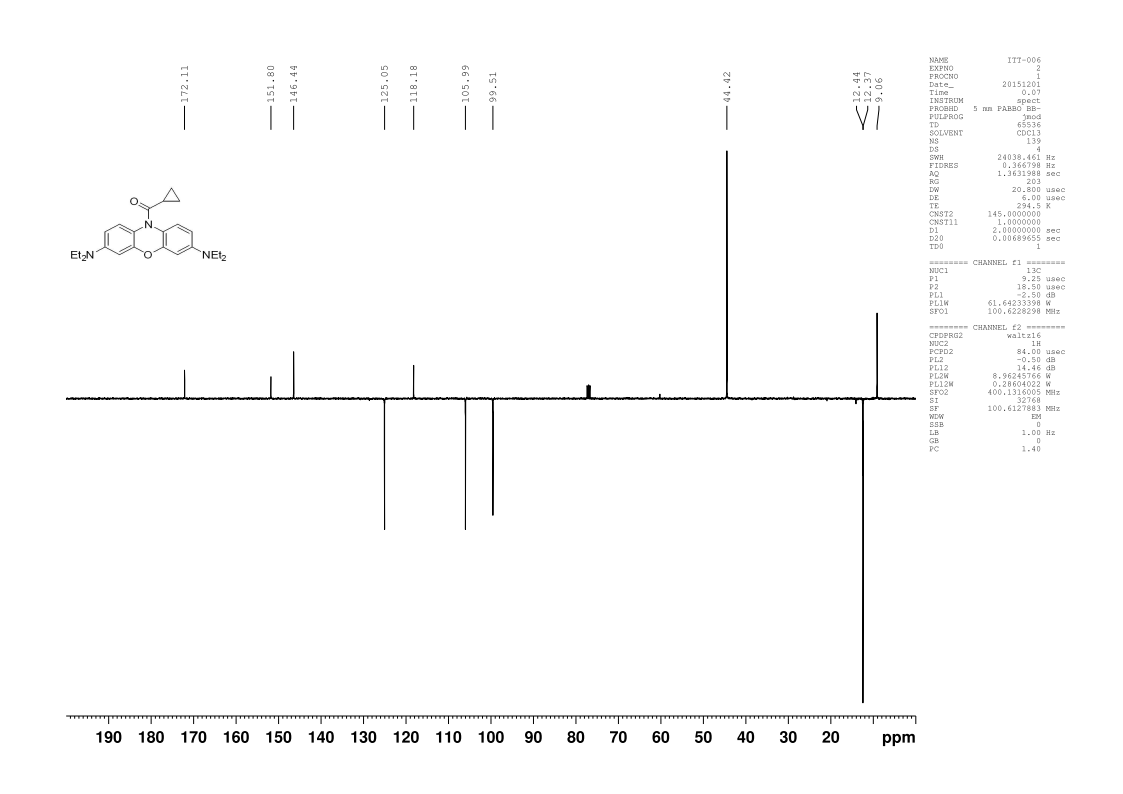


Fig. S4: ^1^H- and ^13^C-NMR Spectra of ITT-004


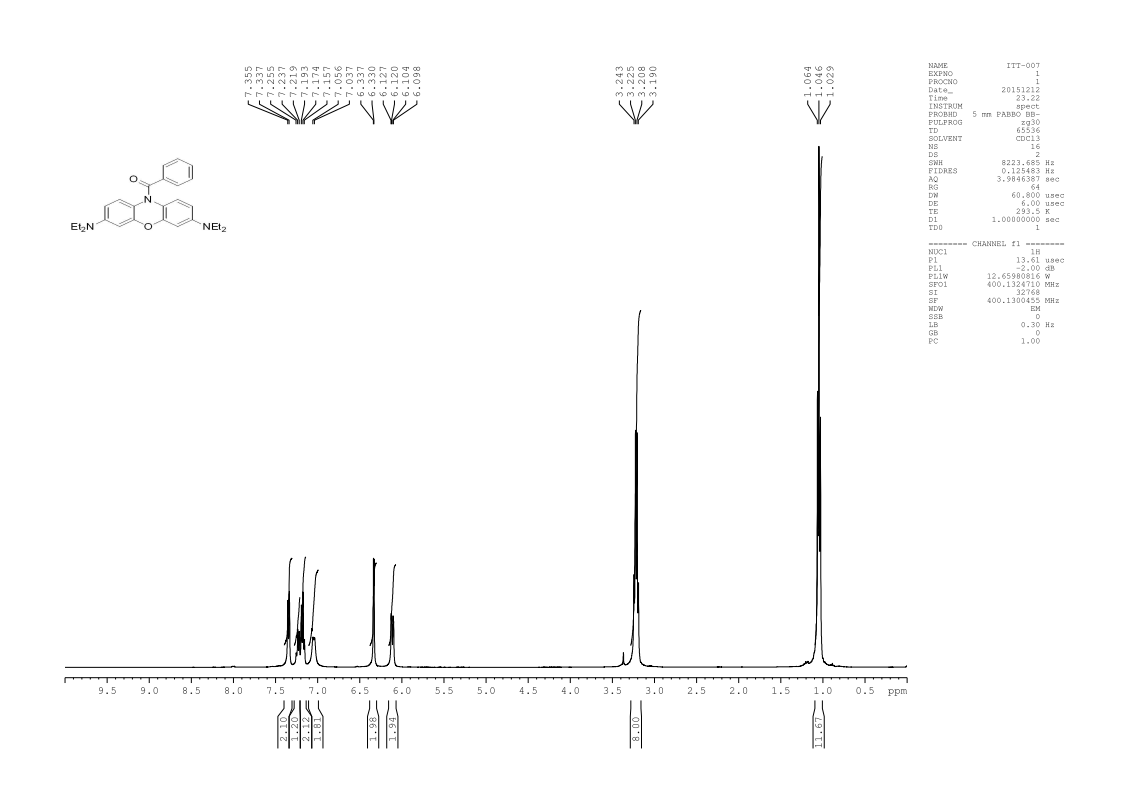

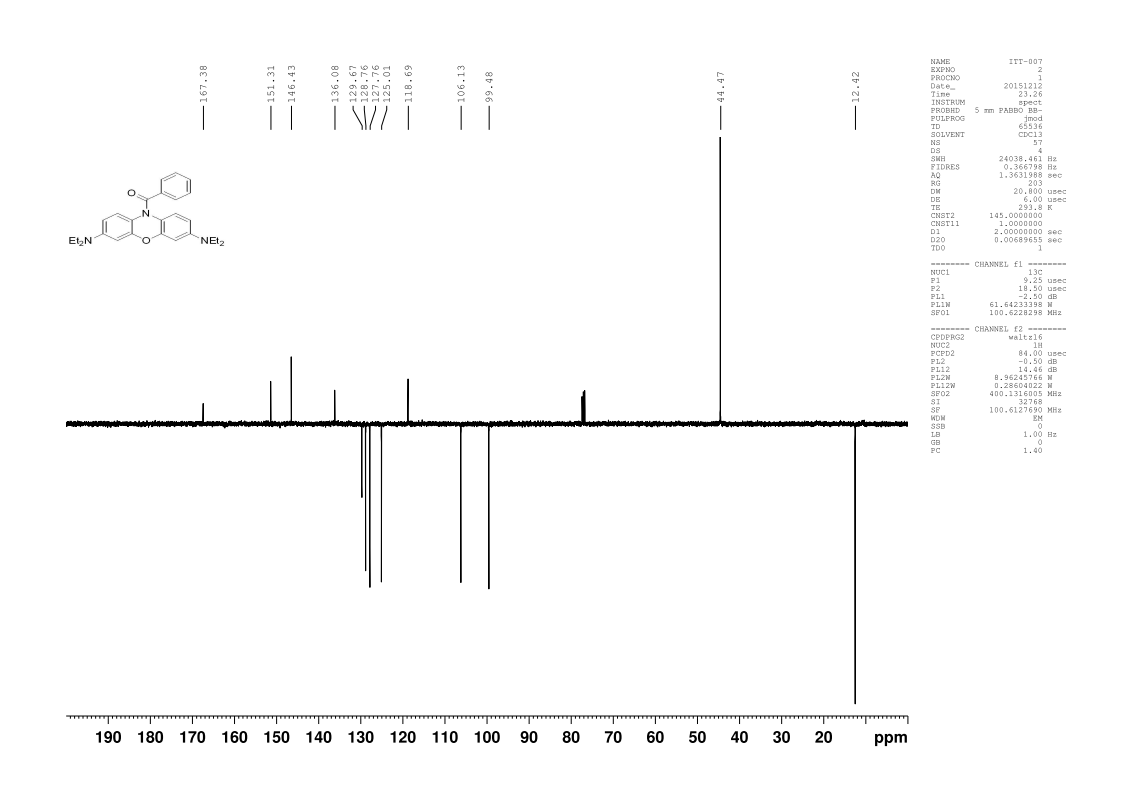


Fig. S5: ^1^H- and ^13^C-NMR Spectra of ITT-005·HCl


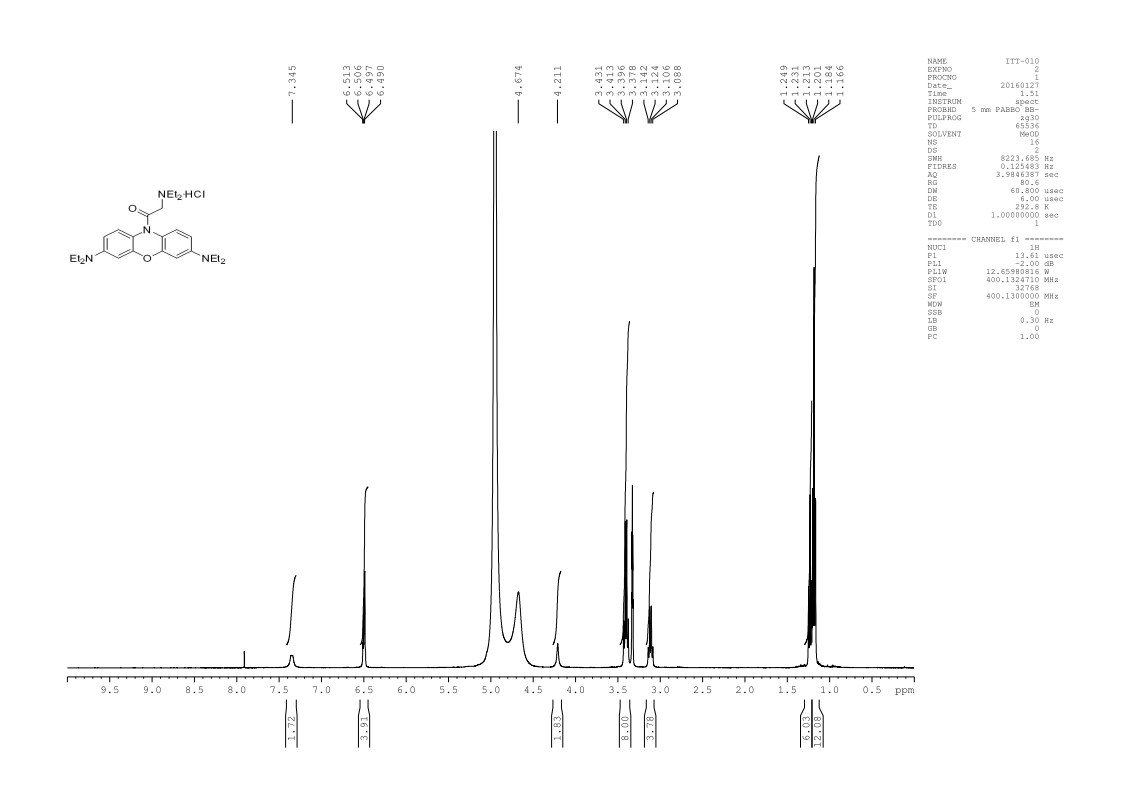

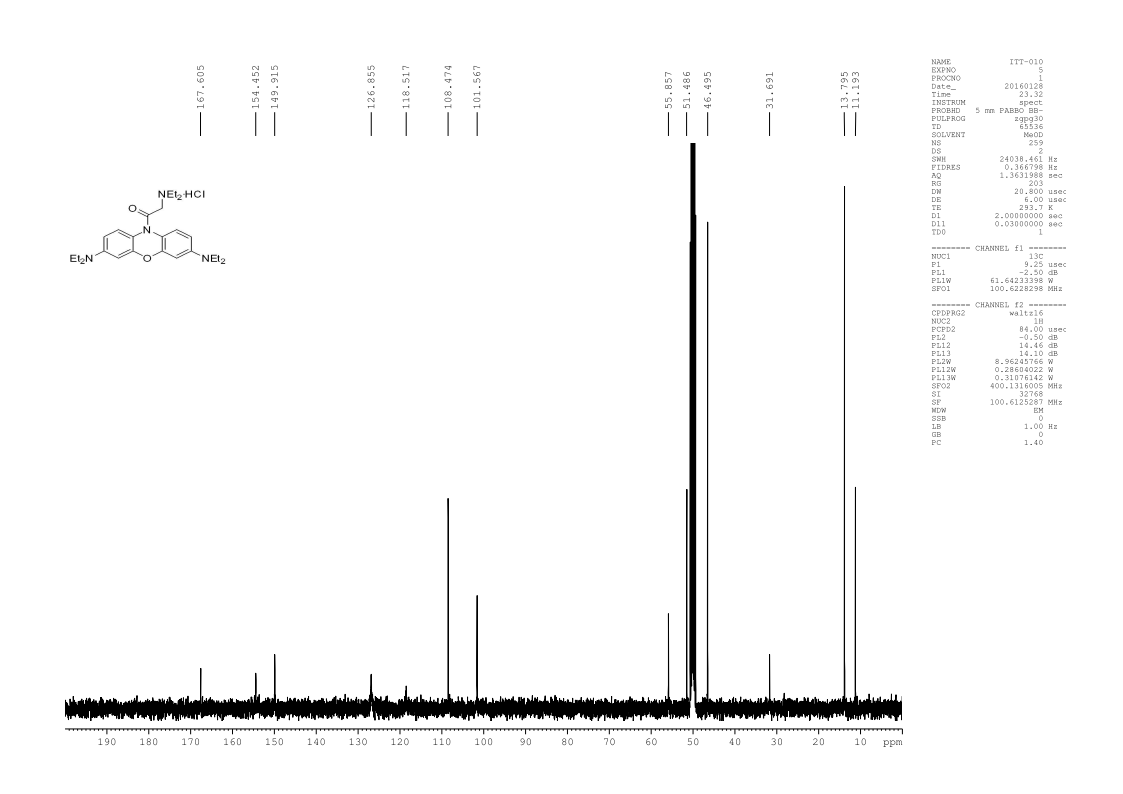


Fig. S6: ^1^H- and ^13^C-NMR Spectra of ITT-006


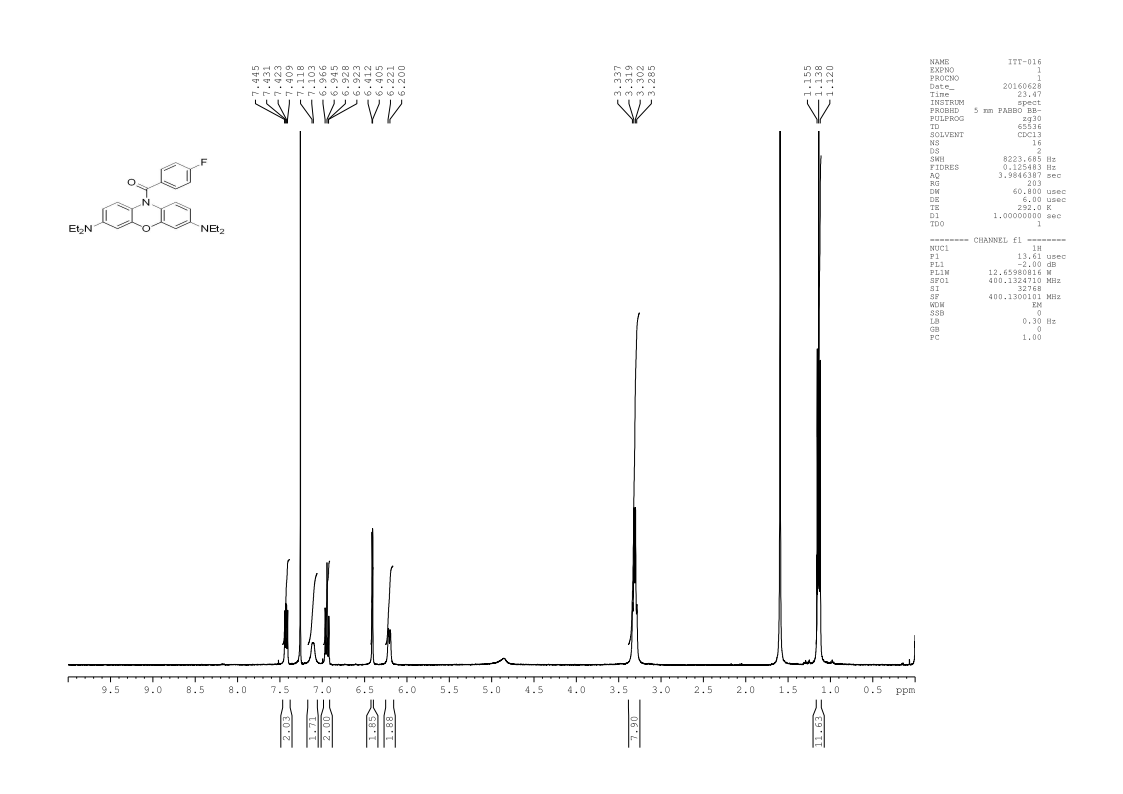

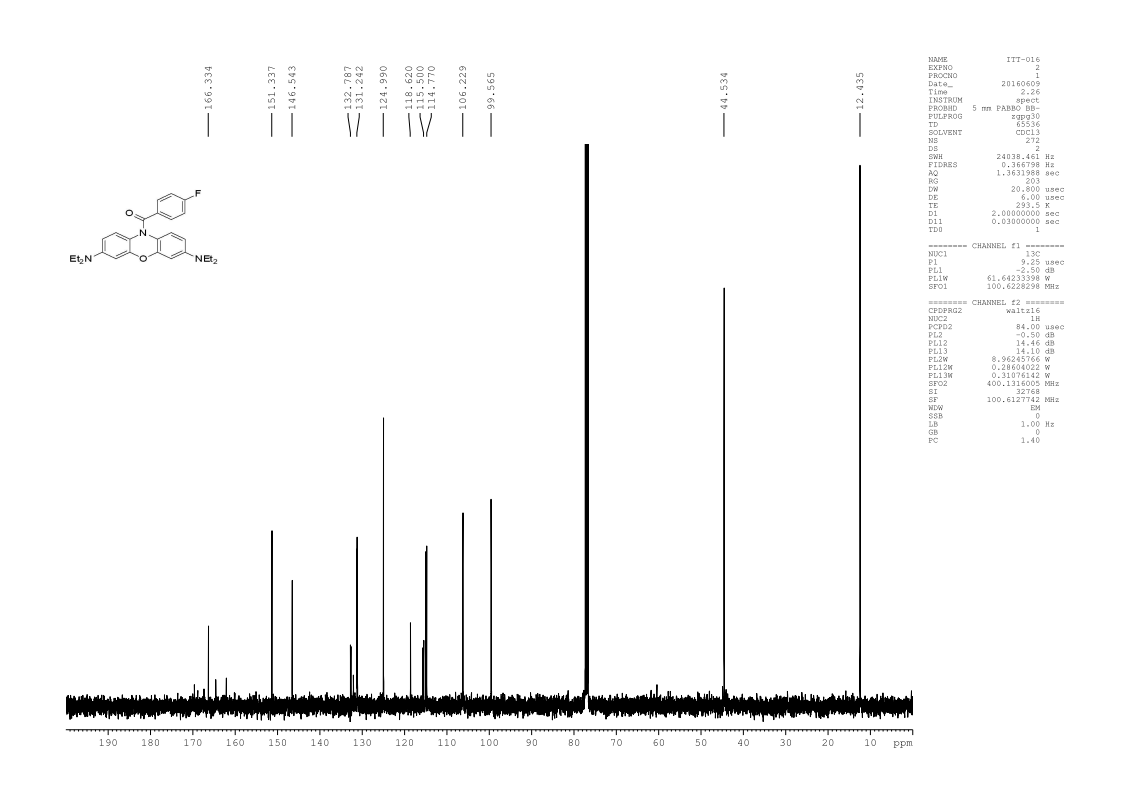


Fig. S7 Kaplan-Meier curve of representative control groups
